# Evidence for the emergence of leg sympathetic vasoconstrictor tone with age in healthy women

**DOI:** 10.14814/phy2.12275

**Published:** 2015-01-27

**Authors:** David J. Moore, Matthew A. Barlow, Joaquin U. Gonzales, Cheri L. McGowan, James A. Pawelczyk, David N. Proctor

**Affiliations:** Noll Laboratory, Department of Kinesiology, The Pennsylvania State University, University Park, Pennsylvania; Intercollege Graduate Degree Program in Physiology, The Pennsylvania State University, University Park, Pennsylvania; Department of Biology, Eastern New Mexico University, Portales, New Mexico; Department of Health, Exercise & Sport Sciences, Texas Tech University, Lubbock, Texas; Department of Kinesiology, University of Windsor, Windsor, Ontario, Canada

**Keywords:** Aging, blood flow, sympathetic, vascular conductance

## Abstract

While muscle sympathetic nerve activity (MSNA) is elevated with advancing age, correlational evidence suggests that, in contrast to men, basal MSNA is not related to resting lower limb hemodynamics in women. However, limited data exists in women that have attempted to *directly* assess the degree of limb sympathetic vasoconstrictor tone, and whether it is altered with age. To address this issue, we measured changes in femoral artery vascular conductance (FVC) during an acute sympatho‐inhibitory stimulus (−60 mm Hg neck suction, NS) in groups of healthy younger (*n* = 8, 23 ± 1 years) and older (*n* = 7, 66 ± 1 years) women. The percent change in FVC in response to NS was significantly augmented in the older (*P* = 0.006 vs. young) women. Although NS caused no significant change (3 ± 3%, *P* = 0.33) in FVC in the young women, there was a robust increase in FVC (21 ± 5%, *P* = 0.003) in the old women. Collectively, these findings provide evidence that in women, leg sympathetic vasoconstrictor tone emerges with age.

## Abstract (precis)

In the present study, we sought to compare the amount of sympathetic vasoconstriction in the resting lower limbs (i.e., legs) of younger and older women. Leg (femoral artery) vascular conductance increased in older but not younger women during an acute sympatho‐inhibitory stimulus. These findings suggest that in women there is an emergence of lower limb sympathetic vasoconstrictor tone with advanced age.

## Introduction

Regulation of the skeletal muscle vasculature plays an important role in systemic blood pressure and metabolic homeostasis (Lind and Lithell 1993). In humans, the lower limbs (i.e., legs) account for ~60% of total skeletal muscle mass (Gallagher et al. 1997). As a result, age‐associated changes in the caliber of skeletal muscle resistance vessels in the lower limbs (Dinenno et al. 1999, 2001a,b; Moreau et al. 2003; Smith et al. 2007) may contribute to the increased prevalence of metabolic and cardiovascular diseases in older adults (Lind and Lithell 1993). Thus, understanding the physiological mechanisms underlying these changes could have therapeutic implications.

Muscle sympathetic nerve activity (MSNA; peroneal nerve) to the leg vasculature becomes elevated with advancing age (Ng et al. 1993; Narkiewicz et al. 2005). In older men, these higher levels of MSNA (compared with young men) ultimately result in an augmented sympathetic vasoconstrictor tone (Dinenno et al. 2001a), even in the presence of blunted *α*‐adrenergic responsiveness (Smith et al. 2007). As a result, MSNA is negatively related to resting leg blood flow and vascular conductance in men. Further, statistically controlling for MSNA in men abolishes the effect of age on these hemodynamic variables (Dinenno et al. 1999).

In contrast to findings in men, age‐group differences in MSNA do not appear to explain attenuated leg blood flow and vascular conductance values observed in older women (Moreau et al. 2003). This null association is interesting considering that women also experience an increase in basal MSNA with age (Ng et al. 1993; Moreau et al. 2003; Barnes et al. 2014), which may exceed that observed in men (Matsukawa et al. 1998; Narkiewicz et al. 2005). However, any potential age‐group and/or inter‐individual differences in either neural (e.g., adrenergic neurotransmitter release or vascular responsiveness) or non‐neural (e.g., resistance vessel structure) factors could contribute to the lack of an observed association in women.

Accordingly, it remains unresolved whether aging influences leg sympathetic vasoconstrictor tone in women. In the present study, we assessed leg (femoral artery) vascular conductance responses to acute sympathetic inhibition with carotid artery baroreceptor loading (−60 mm Hg neck suction) as a measure of whole‐limb sympathetic vasoconstriction in groups of healthy younger and older women.

## Methods

This study and its procedures were approved by the Office for Research Protections at The Pennsylvania State University in agreement with the guidelines set forth by the Declaration of Helsinki.

### Participants

Eight young and nine older women were recruited from the local (Centre County, Pennsylvania) community. Two older women were excluded because of technical errors measuring beat‐to‐beat blood pressure. As a result, data from eight younger (23 ± 1 years, range: 21–30 years) and seven older (66 ± 1 years, range: 61–71 years) women were included in this analysis. Prior to inclusion in this study, all participants provided their written informed consent, and completed a health history questionnaire and physical examination performed by a qualified medical staff member at Penn State's Clinical Research Center. All participants reported no history of cardiovascular, metabolic (e.g., diabetes), or neurological disease. All participants were nonsmokers, nonobese (body mass index <30 kg·m^−2^), and normotensive (seated resting blood pressure <140/90 mm Hg). Individuals were excluded if they had fasted blood concentrations of LDL cholesterol ≥160 mg·dL^−1^ or triglycerides ≥200 mg·dL^−1^, were taking anti‐hypertensive or lipid‐lowering medication, or were currently taking hormone replacement therapy. Younger women were eumenorrheic and not currently taking hormonal contraceptives.

### Cardiorespiratory fitness testing

Cardiorespiratory fitness was determined as the peak rate of pulmonary oxygen consumption (VO_2peak_; TrueMax 2400, Parvo Medics, Sandy, Utah) measured during a modified Balke treadmill test performed until volitional fatigue. After a 2‐min warm up at 2.5 mph, the speed of the treadmill was adjusted to elicit a heart rate that was ~75% of each subject's age‐predicted maximum (Tanaka et al. 2001). Afterward, the incline of the treadmill was increased by 2.5% every 2 min. VO_2peak_ was determined as the highest 30‐sec average achieved during the test.

### Body composition

Whole‐body and regional measures of total mass and lean (fat‐free minus bone) mass were estimated using dual‐energy x‐ray absorptiometry (DEXA; model QDR 4500W, Hologic, Waltham, MA). Estimates of left leg (i.e., the experimental limb) tissue masses were derived from the entire width and length of the lower limb beginning at a cut‐point located mid‐way along the femoral neck.

## Experimental Protocol

### General procedures

Experimental measurements were performed on a separate day from cardiorespiratory fitness testing. Testing in young women was performed between days 1 and 7 of the menstrual cycle to minimize potential variability resulting from fluctuations in circulating estrogen and progesterone. All measurements were performed with participants seated in a semi‐recumbent (~60° from horizontal) position. Throughout the study protocol, beat‐to‐beat blood pressure was collected continuously by means of finger photoplethysmography (Finometer Midi, Finapres Medical Systems, Amsterdam, the Netherlands) with the hand positioned at the heart level. Prior to carotid artery baroreceptor loading, participants sat quietly for a 10–15‐min rest period to allow cardiovascular and hemodynamic variables to achieve a steady baseline. Resting values of heart rate, mean arterial pressure (MAP), and femoral artery blood flow (FBF) and vascular conductance (FVC) were determined from the average of a continuous 2‐min segment at the end of this baseline period.

### Leg hemodynamic measurements

Left common femoral artery diameter (FAD) and blood flow velocity (FFV) were measured using Doppler ultrasound (HDI 5000; Philips, Bothell, WA) with a 6 MHz probe. FAD was determined from a 10–15‐sec recording acquired during baseline rest. Commercially available edge‐detection software (Brachial Analyzer Software, Medical Imaging Applications; Iowa City, IA) was used to calculate the average FAD across the cardiac cycle from this recording. Beat to beat measurements of FFV were acquired at an insonation angle of 60° and a sample volume that encompassed the entire width of the vessel. FBF was calculated using the equation *π* * (FAD/2)^2^ * (FFV) * 60. FVC was calculated as FBF/MAP.

### Responses to carotid artery baroreceptor loading

Participants were fitted with a foam‐lined malleable lead collar that encompassed the anterior two‐thirds of the neck, and formed a tight seal along the mandibles and clavicles. In order to temporarily inhibit sympathetic neural outflow, sustained 5‐sec pulses of neck suction (NS; −60 mm Hg) were delivered during a 10–15‐sec end expiratory breath hold as previously described (Keller et al. 2003; Ogoh et al. 2003). Three to four trials of NS, each separated by at least 30 sec, were performed on all women. These trials were averaged to generate a mean response for MAP, FBF, and FVC for each participant. Beat‐to‐beat changes in MAP in response to NS were determined using commercially available software (WinCPRS, Absolute Aliens Oy, Turku, Finland). The mean FFV across the four cardiac cycles during which MAP was the lowest following NS was used to calculate the peak response for FBF and FVC. Changes in MAP, FBF, and FVC were determined by comparing these values to the mean of the ten cardiac cycles preceding the application of NS. All subjects were familiarized with the NS procedure on a previous visit.

### Statistical analyses

Statistical analyses were performed using Prism 5 software (GraphPad, La Jolla, CA). Significance for all analyses was determined as *P* < 0.05. Data are presented as mean ± SEM unless otherwise specified. Group comparisons of baseline characteristics, resting hemodynamic measurements, and hemodynamic responses to carotid baroreceptor loading were performed using independent *t*‐tests. Absolute and relative responses of hemodynamic variables within each age group were compared against a hypothetical no change (Δ = 0) using 1‐sample *t*‐tests.

## Results

### Subject characteristics

Characteristics of the young and old groups of women are summarized in Table 1. While height and body mass were not different between age groups, whole body and abdominal adiposity were greater in the older women. Peak oxygen uptake (VO_2peak_) normalized to total body mass was lower among older compared with younger women; however, age group differences were no longer present when VO_2peak_ was normalized to lean body mass. Compared with young women, old women had higher fasting total and low‐density lipoprotein cholesterol, but similar fasting high‐density lipoprotein cholesterol, triglycerides, and glucose. There was no difference in either total or lean leg (i.e., experimental limb) mass between young and old women.

**Table 1.** Subject characteristics

	Young women	Old women	*P* value
*n*	8	7	
Age (years)	23 ± 1	66 ± 1	<0.001
Height (cm)	163 ± 2	160 ± 2	0.28
Body mass (kg)	61.4 ± 3.8	67.6 ± 3.4	0.25
Body fat (%)	27 ± 1	38 ± 2	<0.001
Abdominal fat (%)	22 ± 2	36 ± 2	<0.001
VO_2peak_ (mL· 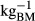 ·min^−1^)	38 ± 2	24 ± 1	<0.001
VO_2peak_ (mL· 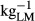 ·min^−1^)	55 ± 3	45 ± 4	0.079
Total cholesterol (mg·dL^−1^)	155 ± 8	208± 7	<0.001
LDL cholesterol (mg·dL^−1^)	82 ± 6	122 ± 12	0.008
HDL cholesterol (mg·dL^−1^)	55 ± 4	63 ± 7	0.33
Triglycerides (mg·dL^−1^)	93 ± 11	115 ± 15	0.27
Glucose (mg·dL^−1^)	87 ± 2	92 ± 3	0.13
Leg mass (kg)^1^	10.4 ± 0.7	10.9 ± 0.7	0.57
Lean leg mass (kg) ^1^	6.7 ± 0.4	6.2 ± 0.2	0.37

Data are presented as Mean ± SEM.

BM, total body mass; LM, lean body mass; LDL, low‐density lipoprotein; HDL, high‐density lipoprotein.

^1^ DEXA‐derived estimates of tissue mass in the experimental (i.e., left) leg.

### Resting hemodynamic measurements

Baseline measurements of resting systemic and leg hemodynamic variables are presented in Table 2. Resting heart rate, femoral artery diameter, and femoral artery flow velocity were not different between young and old women. Accordingly, there was no effect of age on whole limb femoral artery blood flow (FBF). In contrast, mean arterial pressure (MAP) was significantly higher in the group of older women. Therefore, calculated femoral artery vascular conductance (FVC) was on average lower in older women; however, this difference did not reach statistical significance.

**Table 2.** Resting hemodynamic measurements

	Young women	Old women	*P* value
Heart rate (beats·min^−1^)	72 ± 4	64 ± 3	0.17
MAP (mm·Hg)	85 ± 3	99 ± 3	0.011
FAD (mm)	8.0 ± 0.2	7.7 ± 0.2	0.39
FFV (cm·sec^−1^)	5.2 ± 0.3	4.5 ± 0.4	0.34
FBF (mL·min^−1^)	155 ± 16	130 ± 18	0.31
FVC (mL·min^−1^·mm·Hg^−1^)	1.8 ± 0.1	1.3 ± 0.2	0.054

Data are presented as Mean ± SEM.

MAP, mean arterial pressure; FAD, femoral artery diameter; FFV, femoral artery flow velocity; FBF, femoral artery blood flow; FVC, femoral artery vascular conductance.

### Hemodynamic responses to carotid artery baroreceptor loading

**Figure 1.**
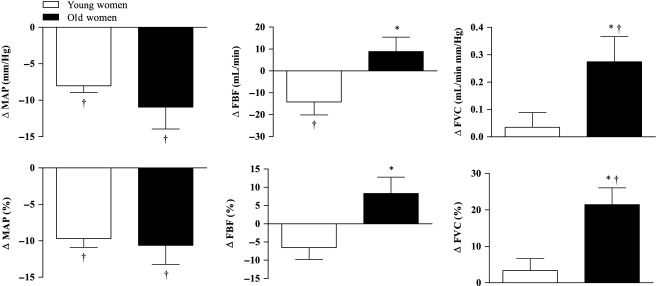
Absolute (top) and relative (bottom) changes in mean arterial pressure (MAP), femoral artery blood flow (FBF), and femoral artery vascular conductance (FVC) in response to carotid baroreceptor loading (−60 mm Hg neck suction) in young and old women. *Signifies different from young women, *P* < 0.05. ^†^Signifies significant change from baseline (i.e., Δ ≠ 0), *P* < 0.05.

Hemodynamic responses to carotid baroreceptor loading with neck suction (NS, −60 mm Hg) are presented in Fig. 1. In response to NS, MAP significantly decreased in both age groups (all *P* < 0.05). The magnitude of the changes in MAP did not differ between the young and old women (∆ MAP: *P* = 0.34, % ∆ MAP: *P* = 0.74). Conversely, FBF responses to NS were dependent on age (∆ FBF: *P* = 0.021, % ∆ FBF: *P* = 0.016). In young women, NS caused a small but statistically significant (*P* = 0.049) decrease in FBF; however, this decrease was no longer significant when expressed as a percentage change (*P* = 0.082). In old women, NS resulted in no significant change in FBF expressed in either absolute (*P* = 0.22) or relative units (*P* = 0.11).

Normalization of FBF responses to the changes in MAP following NS resulted in FVC responses that were also dependent on age (∆ FBF: *P* = 0.038, % ∆ FBF: *P* = 0.006). In young women, NS caused no change in FVC (∆ FVC: *P* = 0.54, % ∆ FVC: *P* = 0.33). In old women, however, FVC was significantly increased (∆ FVC: *P* = 0.025, % ∆ FVC: *P* = 0.003) in response to NS.

## Discussion

The primary novel finding of the present study is that sympathetic vasoconstrictor tone of the lower limb (i.e., leg) is augmented in old compared with young women. Specifically, young women exhibit no detectable sympathetic vasoconstriction in their leg vasculature at rest. Conversely, there appears to be robust vasoconstriction in the resting lower limbs of older women. Collectively, these findings suggest that with advanced age there is an “emergence” of a sympathetic neural influence on leg vascular tone in women. These conclusions are based on the observation that acute sympathetic inhibition via carotid artery baroreceptor loading increased femoral artery vascular conductance (FVC; Fig. 1) ~21% in old women, whereas FVC was unchanged in the younger women with the same intervention.

It is well established that muscle sympathetic nerve activity (MSNA) is augmented with age in women (Ng et al. 1993; Matsukawa et al. 1998; Moreau et al. 2003; Narkiewicz et al. 2005; Hart et al. 2011; Barnes et al. 2014). Yet, whether sympathetic vasomotor tone is altered in the leg skeletal muscle vasculature of older compared with younger women has remained less clear. Findings from previous correlational studies have suggested that, in contrast to men, basal sympathetic neural activity is not a significant predictor of resting leg vascular tone in women (Moreau et al. 2003; Hogarth et al. 2007). Specifically, Moreau et al. (2003) observed no association between MSNA and resting whole‐limb vascular conductance in pre‐ or post‐menopausal women. Similarly, Hogarth and colleagues later observed no relation between MSNA and calf vascular resistance in a sample of predominantly (65%) premenopausal women (Hogarth et al. 2007). However, in neither of these studies was the local or systemic adrenergic environment manipulated. As a result, they may yield little information regarding the overall contribution of the sympathetic nervous system (SNS) to leg vascular conductance in this population.

In the present study, we assessed the local hemodynamic response to a physiological sympatho‐inhibitory stimulus as a more direct measure of whole‐limb sympathetic vasoconstriction. Using this approach, our findings provide further support for those of Moreau and Hogarth that the SNS does not contribute to lower‐limb vascular tone in young premenopausal women. Yet, we also provide evidence to suggest that these conclusions may not be applicable to older postmenopausal women who, in contrast, appear to have augmented sympathetic support of leg vascular resistance. Collectively, our present findings are consistent with recent observations regarding the influence of age on autonomic control of blood pressure in women. For instance, older compared with younger women experience larger reductions in total peripheral resistance (TPR) and mean arterial pressure (MAP) following pharmacological suppression of autonomic neural activity via ganglionic blockade (Barnes et al. 2014). Furthermore, carotid baroreflex (CBR)‐mediated reductions in MAP appear to be driven entirely by bradycardia‐induced alterations in cardiac output in young premenopausal women (Kim et al. 2011). However, Credeur and colleagues recently reported evidence suggesting that the modulation of MAP by the CBR in women becomes increasingly more reliant on changes in vasomotor activity with advanced age (i.e., in older, postmenopausal women) (Credeur et al. 2014).

While age group differences in leg vasoconstrictor tone are likely mediated in part by the age‐associated increase in MSNA, differences between young and old women in the responsiveness of vascular adrenergic receptors may also play a key contributing role. For instance, younger women have enhanced *β*_2_‐adrenergic receptor responsiveness compared with men (Kneale et al. 2000). It is postulated that in younger women, greater *β*_2_‐mediated dilation may partially or fully offset *α*‐adrenergic vasoconstriction. Consistent with this hypothesis, younger women exhibit (1) minimal forearm vasoconstrictor responses to brachial artery infusions of norepinephrine (NE); and (2) no relation between MSNA and TPR (Hart et al. 2011). However, local and systemic blockade of *β*‐adrenergic receptors with propranolol augments the forearm vasoconstrictor response to NE and the relation between MSNA and TPR, respectively, in young women. In contrast to findings in young women, propranolol has no effect on peripheral vascular responses to adrenergic agonists or the association between MSNA and TPR in women who are postmenopausal. Therefore, it appears that *β*_2_‐mediated vasodilation is lost in women after menopause, and may contribute to the development of a pro‐vasoconstrictor state with advancing age in women (Hart et al. 2012). However, *leg* vascular responsiveness to *α*‐ or *β*‐adrenergic agonists has not been studied in young or older women. Thus, whether the changes outlined above underlie the effect of age on leg sympathetic tone observed in the present study remains unknown.

### Experimental considerations

In the present study, we were unable to detect a statistically significant difference in resting FVC between the younger and older women (*P* = 0.054, Table 2). It is possible that the similar lean leg mass observed in the different age groups contributed to this null finding. However, age‐associated reductions in resting leg hemodynamics in women (Moreau et al. 2003) as well as men (Dinenno et al. 1999) have been reported to occur independent of changes of tissue mass. It is also important to note that the magnitude of the difference in FVC between age groups in our present study is consistent with those reported in the literature. For instance, Moreau et al. (2003) reported FVC values that were on average ~22% lower in older compared with younger women. Similarly, we observed here that FVC was ~28% lower in the older group of women. It is likely that the lack of a statistical difference in our present study was related to our relatively small sample size (young: *n* = 8, old: *n* = 7). However, this should not distract from the primary finding in the present study that lower‐limb vasodilator responses to acute sympathetic inhibition were augmented in the older women (Fig. 1).

Additionally, MSNA was not measured in the present study. Therefore, it is possible that inadequate basal sympathetic neural activity may have explained the absence of leg sympathetic vasoconstrictor tone in the younger women. However, several studies report an average MSNA burst frequency of 10–20 bursts per minute in younger women (Ng et al. 1993; Moreau et al. 2003; Narkiewicz et al. 2005; Hart et al. 2009, 2011; Barnes et al. 2014). Importantly, these measurements were performed while subjects were in the supine position, while subjects in our present investigation were studied in a semi‐recumbent position. Consequently, basal MSNA in our present study was likely to be equal or greater to previously reported values as a result of potential cardiopulmonary baroreceptor unloading. Furthermore, blockade of autonomic ganglia (Christou et al. 2005; Barnes et al. 2014) or *α*‐adrenergic receptors (Schmitt et al. 2010) causes a measurable reduction in arterial blood pressure and TPR in young women, suggesting the presence of sympathetic vasoconstrictor tone. While this appears to contradict our present findings in younger women, inconsistencies among studies may be attributable to differences in sympathetic vascular transduction of various vascular beds.

## Conclusions

In conclusion, the results of the present study suggest that advanced age results in the emergence of a functional sympathetic vasoconstrictor tone in the leg vasculature of women. The increased sympathetic vasoconstriction observed in the lower limbs of older women may result from either an age‐associated change in adrenergic responsiveness of the peripheral vasculature and/or increased sympathetic neural activity.

## Acknowledgments

The authors wish to acknowledge Peter Raven for his valuable input on the construction and operation of the variable pressure neck chamber used in the present study for carotid baroreceptor loading.

## Conflict of Interest

None declared.
